# Learning of sameness/difference relationships by honey bees: performance, strategies and ecological context

**DOI:** 10.1016/j.cobeha.2020.05.008

**Published:** 2021-02

**Authors:** Martin Giurfa

**Affiliations:** 1 College of Animal Science (College of Bee Science), Fujian Agriculture and Forestry University, Fuzhou 350002, China; 2 Research Centre on Animal Cognition, Center for Integrative Biology, CNRS, University of Toulouse, F-31062 Toulouse Cedex 09, France; 3 Institut Universitaire de France (IUF), France

## Abstract

Humans and non-human primates learn conceptual relationships such as ‘same’ and ‘different, which have to be encoded independently of the physical nature of objects linked by the relation. Consequently, concepts are associated with high-level cognition and are not expected in an insect brain. Yet, various works have shown that the miniature brain of honey bees also learns the conceptual relationships of sameness and difference and transfers these relationships to novel stimuli. We review evidence about sameness/difference learning in bees and analyze its adaptive value within an ecological context. The experiments reviewed cannot be accounted for by low-level strategies and challenge, therefore, the traditional view attributing supremacy to larger brains when it comes to the elaboration of concepts.

**Current Opinion in Behavioral Sciences** 2021, **37**:1–6

This review comes from a themed issue on **Same-different conceptualization**

Edited by **Jean-Remy Hochmann, Ed Wasserman,** and **Susan Carey**

For a complete overview see the Issue and the Editorial

Available online 8th July 2020

**https://doi.org/10.1016/j.cobeha.2020.05.008**

2352-1546/© 2020 Elsevier Ltd. All rights reserved.

## Introduction

Concepts are considered as ‘the glue that holds our mental life together … in that they tie our past experiences together to our present interactions with the world’ [1]. They are typically associated with high levels of cognitive sophistication and are not expected in an insect brain. Yet, research on honey bee learning has shown that even their miniature brains, with only one cubic millimeter and less than one million neurons, can learn conceptual relationships as defined in concept-learning protocols commonly used in vertebrates, thus raising the question of the across-species generality of concept learning and of the minimal neural circuitry necessary to mediate it.

Experiments on concept learning in bees have taken advantage of the easiness with which individually marked bees can be trained to fly to an experimental site where they are rewarded with a drop of sugar water for correct choices of specific visual targets [2, 3, 4]. Interest on the bees’ capacity to master concept learning started with experiments on sameness and difference learning at the beginning of the 2000’s [5]. In this, and in other works that were performed later, the focus was on relational learning, that is, the capacity to extract relationships encoded independently of the physical nature of the objects linked by the relation (e.g. ‘same as’, ‘different from’) [6, 7, 8, 9, 10]. Acquisition of relational learning is evaluated in a transfer test, in which subjects are presented with novel stimuli. Performance in this test reveals whether they have extracted a relation that is applied, irrespective of the novelty of the situation [10]. Here, we review evidence supporting the existence of relational-concept learning in honey bees focusing on sameness/difference learning. Furthermore, we discuss the meaning of 'concept learning’ in bees and its adaptive value in an ecological context.

## Conceptual learning of sameness/difference relationships

**Figure 1:**
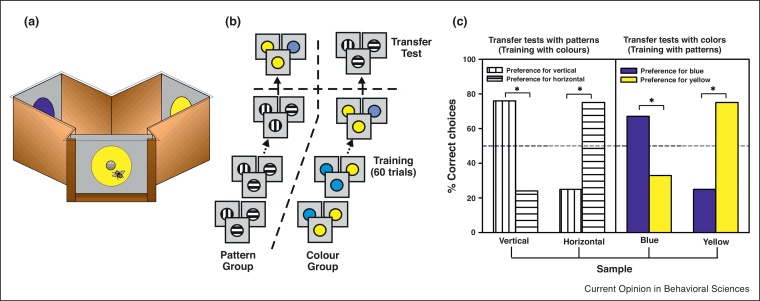
Sameness learning in honey bees [5]. **(a)** Y-maze used to train bees in a delayed matching-to-sample task. Bees entered into the maze to collect sugar solution on one of the back walls of the maze. A sample was shown at the entrance before bees accessed the arms of the maze. **(b)** Training protocol. A group of bees were trained during 60 trials with black-and-white, vertical and horizontal gratings (Pattern Group); another group was trained with colors, blue and yellow (Color Group). After training both groups were subjected to a transfer test with novel stimuli (patterns for bees trained with colors, colors for bees trained with patterns). **(c)** Performance of the Pattern and the Color Group in the transfer tests. Both groups chose the novel stimulus corresponding to the sample although they had no experience with such test stimuli.

The first study on conceptual learning in honey bees addressed the question of sameness and difference learning in bees [5], using the protocols of delayed matching to sample (DMTS) and delayed non-matching to sample (DNMTS), respectively. Bees were trained to enter a Y-maze to collect sucrose solution on one of the arms of the maze (Figure 1a). After being presented with a sample stimulus at the entrance of the maze, the bee had to choose between two target stimuli displayed on the maze arms, one was identical to the sample and the other was different. In sameness learning, choice of the identical stimulus was rewarded. In difference learning, choice of the different stimulus was rewarded. In both cases, the position of the reward changed randomly between the arms of the maze from visit to visit. In a first experiment, individually marked bees were trained to determine whether they could learn a concept of sameness. Bees were presented with a changing non-rewarded sample (i.e. one of two different color disks — ‘color group’ — or one of two different black-and-white gratings, vertical or horizontal — ‘pattern group’) at the entrance of a maze (Figure 1b). The bee was rewarded only if it chose the stimulus identical to the sample displayed at the entrance of the maze. Bees trained with colors and presented in transfer tests with vertical or horizontal black-and-white gratings that they had not experienced before, chose the grating identical to the sample at the entrance of the maze. Similarly, bees trained with the gratings and tested with colors in transfer tests chose the novel color corresponding to that of the sample (Figure 1c). Transfer was not limited to different types of visual stimuli (pattern versus color), but occurred also between odors and colors [5]. Bees also mastered a DNMTS task, thus showing that they learn the concept of difference between stimuli as well [5].

Sameness learning was later confirmed in various studies that used the DMTS task to address different questions in the visual domain. One of them aimed at characterizing the duration of the memory established for the sample seen at the entrance of the maze. To this end, the path between the sample and the decision chamber from where the two alternatives to be chosen were visible was progressively extended. The sample memory lasts for approximately five seconds [11], a period that coincides with the duration of other visual and olfactory short-term memories characterized in bees [12]. DMTS was also used in categorization experiments aimed at studying the capacity of bees to group images according to broad classes such as ‘radial flower’, ‘plant stems’ or ‘landscapes’ [13].

Recently, experiments on sameness/difference learning in bumblebees were reported [14], yet with a questionable methodology. Discussing this aspect is important to realize which mistakes need to be avoided in research on concept learning by insects. In this work, choices occurring within an experimental arena displaying visual stimuli were recorded but instead of focusing on individual performances, the whole colony could access the arena, so that at any moment, many bees were flying inside it. This methodology makes impossible to assess, not only an individual’s contribution to the cumulative choices recorded, but also the statistical soundness of the conclusions, as independent and dependent data were combined in an indiscriminate way [14]. Learning being an individual’s property, its recording and analysis should be performed at the individual level.

## Conceptual learning of oddity and non-oddity

Oddity and non-oddity learning refers to the capacity to learn ‘odd versus even’ based on a variable set of stimuli. In the basic form of oddity learning, an animal may learn to choose one stimulus that is different from two identical stimuli. In subsequent trials, stimuli are varied but the relation that opposes odd versus even is maintained. In non-oddity learning, the animal is rewarded for choosing the even stimuli, that is, the one identical to another stimulus, and not the odd one. As in the previous experiments, transfer tests have to be performed to determine whether the experimental subjects have learned a conceptual relationship. Thus, oddity and non-oddity learning resemble in several ways to difference and sameness learning, respectively, as they may access similar representations of stimulus identity: ‘odd’ may rely on a concept of difference while ‘even’ may rely on a concept of ‘sameness’.

Experiments on oddity and non-oddity learning in free-flying bees were performed [15^•^], yet using a different methodology compared to that of the previous work on sameness/difference learning mentioned above [5]. While the latter used the DMTS and DNMTS tasks, that is, delayed stimulus presentation with respect to a sample stimulus, the oddity/non-oddity experiments presented always three stimuli simultaneously. Bees were trained to choose among three colored discs presented horizontally [15^•^]. Two were identical and one was different. Stimuli changed from visit to visit, keeping the odd versus even relationship constant. Some bees were rewarded for choosing based on the odd concept while others were rewarded for choosing based on the even concept. Bees learned the discrimination task and transferred correctly their choice to either novel odd or even stimuli depending on the training received [15^•^].

In addition to the similar relational representations that bees may use during sameness/difference learning and non-oddity/oddity learning, they could rely on an additional type of representation, in particular when stimuli are presented simultaneously. As bees possess a proven sense of numerosity (see below and [16]), another way to solve the oddity/non-oddity discrimination could be to use the numerical relationship of 1 versus 2, as these two quantities are learned in absolute terms by the bees, that is, irrespective of variations in the physical quality of the items displayed [17^•^]. Further experiments should unravel the nature of the representations used in these discriminations.

## Sameness learning in numerosity judgments

**Figure 2:**
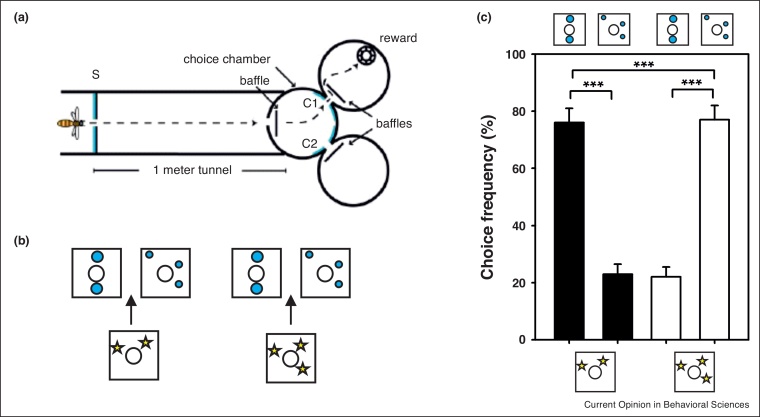
Sameness learning in numerosity judgments by honey bees [19]. **(a)** Bees were trained to fly into a tunnel using a delayed matching-to-sample protocol in which they had to match stimuli containing two or three elements. The sample with two or three elements was shown at the maze entrance (in blue) before bees accessed a decision chamber displaying two stimuli (C1, C2). Only the stimulus showing the same number of items as the sample allowed access to a feeder, which was hidden by a baffle. **(b)** The bees had to choose the arm containing the stimulus composed of the same number of elements as the sample to obtain sucrose reward. The appearance of the elements and their spatial positions differed between the sample and the target so that bees had to focus on number and not on other perceptual cues to solve the task. **(c)** In transfer tests, the bees were able to match the stimuli according to the number of their composing elements, if numbers did not exceed four. Modified from Ref. [19].

Numerosity judgments in bees have been intensively explored in the last few years and several experiments have shown a capacity to use relative and absolute numerosity in different experimental contexts [16,17^•^,^18^]. In one of the first studies on the numeric sense of bees, sameness learning was used as bees were trained in a DMTS task to match visual stimuli based on their number [19]. Free-flying bees were trained to fly into a tunnel displaying an array of items at the entrance (Figure 2a). Entering the tunnel allowed them to access a decision chamber where two item arrays were visible. The bees had to match their choice to the stimulus containing the same number of items as the sample presented at the maze entrance (Figure 2b). Bees trained to match sample arrays with two or three items learned the task and transferred correctly their choice to novel arrays with the appropriate number of items but differing in color, shape and configuration. However, they failed sometimes when the sample contained four items. Higher numbers resulted in more unsuccessful performances, thus suggesting that the limit of numerosity in these experiments may be between 4 and 5 [19]. This work thus used the capacity of bees to learn sameness relationships to uncover basic aspects of their numeric sense. Further works deepened the analysis of the bees’ numeric competences [16,18], yet without using the sameness/difference relationship.

## What do we mean by ‘concept learning’ in bees?

These findings raise the fundamental question of whether the nature of concepts elaborated by bees is comparable to that of humans. Operationally, bees fulfill the experimental criteria used for demonstrating concept learning in vertebrates. They choose based on relationships between stimuli that are variable and whose physical nature is irrelevant for problem solving, and they transfer their choice to novel situations if the learned relationships are preserved. An explanation based on simple associative learning is insufficient to account for this performance as such learning would be stimulus specific and would preclude transfer to unknown stimuli, particularly in the very first experience with them [20].

Further explanations have been proposed to dismiss a conceptual content in this form of problem solving by bees. For instance, their sameness learning was said to rely on their well-known ‘flower constancy’ [21], that is, the fact that a foraging bee stays faithful to the same flower species, as long as it remains profitable in terms of nectar or pollen content [22]. Sameness learning — it was said — may be driven by the tendency to choose the same stimulus in a strategy akin to flower constancy [21]. Yet this explanation ignores the basic fact that flower constancy is a case of associative learning as it relies on obtaining food reward on an exploited flower. The experience of single appetitive reward triggers specific memory phases, with different molecular bases and stability, which drive honey bee choices after leaving the rewarded flower [12,23]. The flower-constancy scenario is, therefore, strictly dependent on the presence of reward, specific for the stimulus learned (i.e. it does not account for stimulus transfer) and is, therefore, not applicable to a sameness protocol in which the animal does not get any reinforcement on the sample that needs to be matched. It explains even less the case of difference learning, where animals have to choose the stimulus that is different from a non-reinforced sample.

Arguments trying to dismiss conceptual learning in bees fail to explain performance in transfer tests, in particular if they involved stimuli form different modalities. Several lines of evidence show that bees access an abstract representation of sameness as they apply this relation not only to colors, gratings or smells, but also to numerosities. In addition, the use of low-level cues was explicitly excluded in most of these experiments. Thus, concluding that bees use concepts to solve discrimination problems provides an appropriate account of what bees do, irrespective of the nature of their conceptual knowledge.

## What do bees gain from concept learning? An ecological scenario for concept learning in bees

The life style of honey bees integrates efficient learning and memory capabilities with sophisticated navigation skills [24] and a flexible visual system for pattern recognition [4]. Together, these capacities may be important for concept learning. This suggestion does not imply that concept learning should be restricted to animals that ally these capacities, but rather that they are cornerstones onto which concept learning might develop.

Honey bees are central place foragers (i.e. their foraging trips start and end at the same fixed point in space: the hive) and are endowed with flexible strategies allowing them to learn and memorize visual patterns both at flowers and at the nest [2]. Visual pattern recognition in bees is highly flexible as it allows extracting relevant features of images and combine them in specific configural representations that may be common to various different stimuli [25,26]. Bees can flexibly generalize their choice to visual images sharing such configurations despite drastic variations in other spatial details and positioning in the visual field [13]. These capacities may intervene in the extraction and recognition of relational concepts, where the focus of attention is the relationship between visual features.

In their foraging bouts, bees use not only sky-based cues but also prominent landmarks and landscape information [27,28]. In this context, extracting relationships such as ‘same’ or ‘different’ may help maintaining routes in a changing and complex environment, where variations in the aspect of landmarks may be otherwise disturbing. These multiple factors, which are the basis of honey bee foraging, may have been determinant for the development of their concept-learning abilities.

In experiments in which free-flying bees are trained to solve visual discriminations such as the ones reviewed here, it is not possible to control for the previous experiences of the animals used. These individuals are foragers (older bees), which may have acquired an extensive prior experience in the field based on intensive foraging and navigation activities. It is thus possible that these bees use pre-existent representations in a way relevant to the tasks trained. Yet, learning curves obtained for some of these tasks do not reveal a bias in the initial learning trials in which choice remains random. This does not exclude that during learning pre-existing representations facilitate problem acquisition.

## Concluding remarks

Concept learning, considered as a cornerstone of human cognition [1,8,29], is a capacity that is now well documented in honey bees. The fact that only these insects have been shown to learn sameness/difference concepts does not make of them a cognitive exception among insects. Other social insect species with efficient memory systems, flexible visual pattern recognition strategies and intense and frequent central-place navigation activities in structured environments, may perhaps be capable of comparable performances.

The case of honey bees reveals that minimal neural architectures are capable of extracting the regularities underlying concept formation. In the absence of a prefrontal cortex, structures that are simpler in terms of their number of neurons, but not necessarily in terms of their functional principles, can support conceptual knowledge in the bee brain. The essential task for the future is to identify the neural circuits that mediate concept learning in the bee brain.

An interesting approach in this direction was achieved by a modeling study that proposed a neural network capable of reproducing the sameness/difference performances found in honey bees [30^••^]. The model focused on a key structure of the bee brain, the mushroom bodies, which have been historically associated with memory storage and retrieval in the case of simple learning forms [31], but also which has been also related with conceptual learning [32]. Mushroom bodies are multimodal integration centers that receive segregated input from different processing areas of the bee brain. They are integrated by thousands of Kenyon cells, which receive this information and further convey it to extrinsic neurons that connect to pre-motor centers. In addition, different types of feedback neurons provide inhibitory signals to the mushroom bodies, either from the output to the input region, or from the output to the output region itself. These neural elements were integrated into a realistic neural architecture that incorporated some of their specific response properties. The resulting neural network was able to reproduce the performance of bees in [5], both in the case of sameness and difference learning [30^••^]. Moreover, it could also reproduce successfully other forms of associative learning demonstrated in honey bees. These results are particularly attractive as they show that a simple neural architecture existing in the bee brain can account for sameness and difference learning. It provides, therefore, cues for identifying comparable architectures in larger brains capable of such conceptual learning. The honey bee has reaffirmed its model status for behavioral studies on concept learning; it should also play a significant role in unraveling the neural bases of this capacity.

## Conflict of interest statement

Nothing declared.

## References and recommended reading

Papers of particular interest, published within the period of review, have been highlighted as:

• of special interest
•• of outstanding interest

## CRediT authorship contribution statement

**Martin Giurfa:** Conceptualization, Writing - original draft, Writing - review & editing, Funding acquisition.
